# Legacy of wood charcoal production on subalpine forest structure and species composition

**DOI:** 10.1007/s13280-022-01750-y

**Published:** 2022-06-09

**Authors:** Matteo Garbarino, Donato Morresi, Fabio Meloni, Nicolò Anselmetto, Flavio Ruffinatto, Massimo Bocca

**Affiliations:** 1 Department of Agricultural, Forest and Food Sciences (DISAFA), University of Torino, Largo Paolo Braccini 2, 10095 Grugliasco, TO Italy; 2 Mont Avic Natural Park, Località Fabbrica 164, 11020 Champdepraz, AO Italy

**Keywords:** Anthracology, Historical ecology, Land-use legacy, LiDAR, Relic charcoal hearths, Spatial patterns

## Abstract

Land-use legacy on forest dynamics at both stand and landscape scale can last for centuries, affecting forest structure and species composition. We aimed to disentangle the history of the charcoal production legacies that historically shaped Mont Avic Natural Park (Aosta Valley, Italy) forests by integrating LiDAR, GIS, anthracological, and field data at the landscape scale. We adopted different geostatistical tools to relate geographic layers from various data sources. The overexploitation due to intensive charcoal production to fuel mining activities shaped the current forests by homogenising their structure and species composition into dense and young stands with a reduction in late seral species such as Norway spruce (*Picea abies*) and an increase in pioneer species such as Mountain pine (*Pinus uncinata*). The multidisciplinary and multi-scale framework adopted in this study stresses the role of historical landscape ecology in evaluating ecosystem resilience to past anthropogenic disturbances.

**Supplementary Information:**

The online version contains supplementary material available at 10.1007/s13280-022-01750-y.

## Introduction

Forest landscapes are complex systems that must be assessed by considering the long-lasting effects of past human activities, so-called land-use legacies (Tappeiner et al. 2020). Landscape history does not just explain the current configuration of forests but guides and influences the future ecosystem resilience to disturbance, management, and global change (Garbarino and Weisberg 2020). Reconstructing the impact of land-use legacies on human-dominated forest landscapes requires a multidisciplinary approach, spanning from historical sciences to ecological tools (Gimmi and Bugmann 2013). Historical records of human impact (e.g. silviculture, grazing, litter collection, burning) on forest structure and composition are often scarce and heterogeneous. Yet some activities, such as charcoal production, introduced pervasive and long-lasting topographic features into the landscape. These archaeological remains, called relic charcoal hearths or simply charcoal kiln sites, are the remaining platforms created as a base to build the charcoal kilns (Young et al. 1996). Charcoal hearths are abundant in historically human-dominated forest landscapes in Europe (Pèlachs et al. 2009; Ludemann 2010) and eastern USA (Young et al. 1996; Raab et al. 2017) and are less documented but present in other countries such as Brazil and Japan (Asada et al. 2002; Bailis et al. 2013).

Charcoal production was one of the most significant preindustrial/industrial forms of forest exploitation in Europe (Ludemann 2010). Relic charcoal hearths are usually abundant near former metal mines because a traditional societal driver of massive charcoal production from woods was the need for archaeo-metallurgy, i.e. Etruscan and Greek, for a lightweight fuel to feed blast furnaces (Di Bérenger 1859). Preindustrial exploitation in Europe took place mostly on marginal forested lands corresponding to late-settled mountainous areas during the Middle Ages, increasing almost steadily for years, reaching a peak in Europe at the end of 1700 before the arising of fossil fuel (Ludemann 2010). For instance, wood charcoal production was significant in France and Germany (Ludemann 2010), Poland (Rutkiewicz et al. 2017), Belgium and Netherlands (Deforce et al. 2021), and Spain (Pèlachs et al. 2009). In the Industrial Age, production of wood charcoal continued in some European regions, such as central Italy, as an energy source for small blast furnaces, heating, and cooking till 1960 (Arrigoni et al. 1985; Carrari et al. 2016). Wood charcoal production was predominantly located in mountainous landscapes under forest cover (Nelle 2003), especially in remote and marginal mountain areas such as Alpine landscapes with low pasture suitability. Forests densely covered these marginal lands, but charcoal production sites were mainly associated with broadleaf-dominated stands (Backmeroff 2001; Carrari et al. 2016; Biondi et al. 2021).

Charcoal remains and relic charcoal hearths are primary data sources to reconstruct forest landscape history. Species classification of native wood charcoal is a fundamental and accurate data source to shed light on the ancient composition of forest species (Ludemann et al. 2017; Máliš et al. 2021). Wood charcoal classification is often associated with radiocarbon dating to reconstruct forest species composition along a temporal gradient (Ludemann et al. 2004). Relic charcoal hearths’ location, shape, and architecture provide fine-scale spatially explicit information on ancient forest management (Máliš et al. 2021; Johnson and Ouimet 2021). Nowadays, technological and methodological evolutions in active optical sensors-based remote sensing, such as laser scanning data (LiDAR), allow the detection of archaeological features hidden by the current forest cover, such as stone walls and relic charcoal hearths (Johnson and Ouimet 2021). Object-based image analysis coupled with machine learning classifiers is a practical analytical tool to identify archaeological features on high spatial resolution Digital Elevation Models derived from aerial laser scanner surveys (e.g. Davis 2019).

Nevertheless, reconstructing historical forest land-use dynamics is often achieved by adopting discipline-specific approaches, such as those common in soil-vegetation relationships (Young et al. 1996), anthracology (Nelle 2003), or remote sensing (Witharana et al. 2018). Anthropogenic disturbances modify the horizontal and vertical structure and the species composition of forest ecosystems, thus, masking natural patterns and processes at different spatial and temporal scales. Therefore, a multidisciplinary approach that integrates various tools and data sources is necessary to analyse social-ecological systems (Beller et al. 2017; Tolksdorf et al. 2020).

This study aims to disentangle the history of wood charcoal production in an inner alpine watershed of the Italian Alps and its effects on the forest structure and composition. We pursued this general objective by integrating different data sources and tools. Specifically, we analysed data from field surveys and airborne LiDAR using dendrometrical, anthracological, and geostatistical tools.

Our research questions were (i) how are relic charcoal hearths distributed, and what geometric characteristics do they have? (ii) how has the legacy of charcoal production changed forest composition during the last 250 years? (iii) what is the influence of natural and anthropogenic drivers on the current forest structure?

We tried to answer these questions in an alpine valley characterised by a complex topography and limited agricultural and pastoral historical land uses.

## Materials and methods

### Study area

**Fig. 1:**
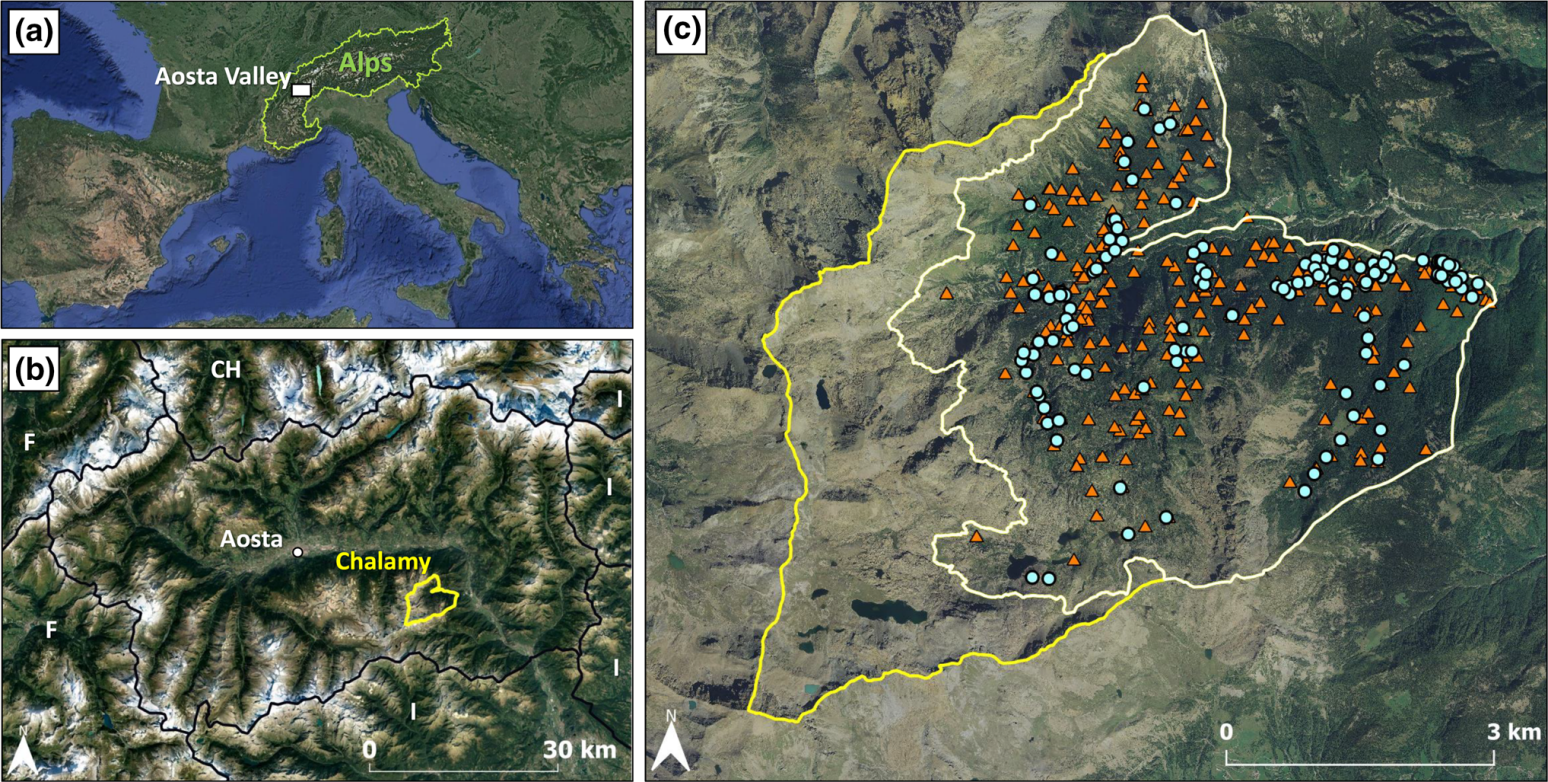
(**a**) Location of the Aosta Valley region within the perimeter of the Alpine Convention. (**b**) Location of the Chalamy valley (yellow line) within the Aosta Valley region. (**c**) Location of the study area (cream line) within the Mont Avic Natural Park (yellow line); sampling design of field surveys for forest structure plots (orange triangular dots) and relic charcoal hearths (RCHs—light blue)

The research area consists of a portion of the Chalamy Valley (45° 40′ 12″ N, 7° 36′ 00″ E) included within the borders of the Mont Avic Natural Park (Italian Graian Alps, Aosta Valley), a protected area since 1989 and included in the EU Natura 2000 Network (IT1202000). The Mont Avic study area occupies a surface area of 2460 ha, the elevation ranges between 920 and 2500 m a.s.l., and steep slopes dominate the watershed (Fig. 1). Specifically, the mean slope within the study area is equal to 27.7° (± 12.3 standard deviation). Mafic and ultramafic rocks of the “Mont Avic Ophiolitic Complex” dominate the valley, where serpentinite is associated with chlorite schists, metagabbros, prasinites, and amphibolites and composes the main lithology (D’Amico et al. 2008). Mean annual temperature ranges from 1 to 3 °C, and precipitations range from 800 to 1200 mm per year, with maxima in spring and autumn. The forest occupies 1600 ha and is dominated by mountain pine (*Pinus mugo* subsp. *uncinata*; 69%), European larch (*Larix decidua*; 15%), and Scots pine (*Pinus sylvestris*; 10%), but European beech dominates at lower elevations in the southeastern part of the watershed (Cremonese et al. 2007).

### Forest history of Mont Avic

A population peak (110 000) during the eighteenth and nineteenth centuries involved a consequent expansion of agriculture and pasture to the detriment of forest cover in the Aosta Valley (Barbieri 1997). Human rural activities were mainly on warmer and gentler slopes, but marginal and inhospitable areas such as Mont Avic likely maintained a diverse and dense forest cover. The great plague of 1630 and the Little Ice Age (LIA) around 1550–1850 caused a general decline in agriculture, but during the period between 1650 and 1750, a substantial increase in mining activities (mainly iron and copper) indirectly affected the forest cover and its structure (Giordano 1864). The so-called “sylvenoire” (black forests), north-facing evergreen conifer forests dominated by Norway spruce (*Picea abies*), were severely harvested to produce charcoal to be used as the primary fuel for blast furnaces for smelting iron and copper materials (Barbieri 1997). Two major blast furnaces sites are still visible at Mont Avic: (i) *La Servaz* that actively smelted magnetite coming from *Lac Gelé* mine in the period 1694–1828 and (ii) *Perrot* that actively smelted copper coming from *Herin* mine starting between the end of the fourteenth century and the beginning of fifteenth century until 1792, and iron until 1821 (Castello and Paganone 2016; Castello 2020–2021). The mixed conifer high forest was managed with a silvicultural system based on short-rotation clearcuts, i.e. 30–40 years, because of the need for small pieces of wood (Barbieri 1997).

For this reason, nowadays, relic charcoal hearths (RCHs) are still present under the forest canopy as a cultural heritage of the charcoal-making process. Small entity thinning and cleaning sylvicultural operations characterise the recent (1940–2021) period as registered by the Verres municipality’s forest management plan (1992). Goat grazing was reported in 1950–1980 with a limited influence at the watershed scale. Nowadays, the human pressure is limited to tourist trekking and extensive grazing at Pra Oursie pasture. Post-abandonment forest gain and climate change are leading to a simplification of lower elevations and a fragmentation of the upper belt (Anselmetto et al. 2021).

### Relic charcoal hearths remote sensing detection

**Fig. 2:**
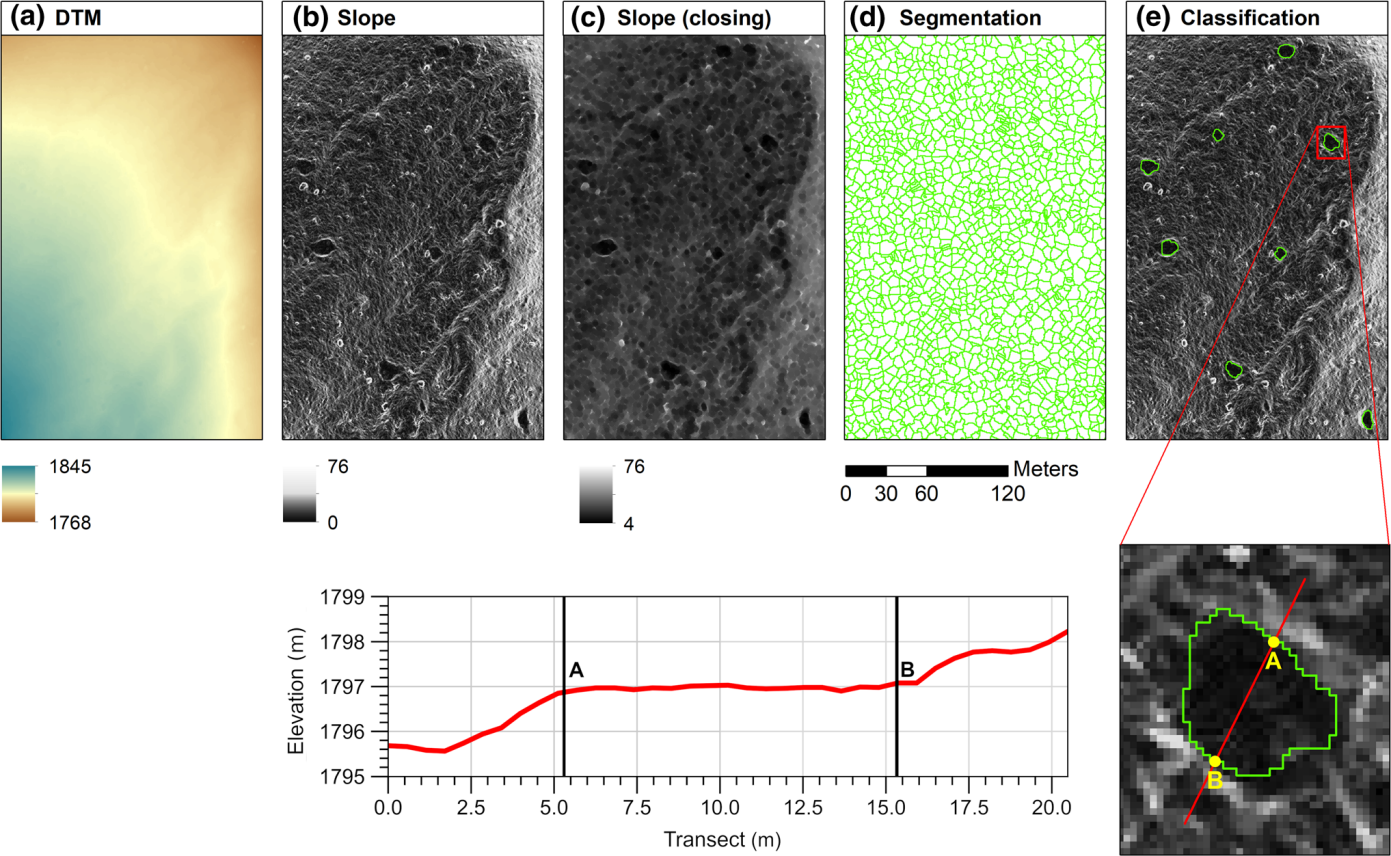
Workflow adopted at Mont Avic to map RCHs: **a** digital elevation model at 0.5 m resolution, **b** slope map, **c** slope map filtered by using the closing procedure, **d** image segmentation, **e** classification of the segmented objects. Bottom-right part of the figure: an example of RCH recognisable in the slope raster derived from the DTM: both the central flat platform (black) and the edges with high local slope (grey) are distinguishable; the red line transect crossing the RCH is oriented towards the maximum slope direction. Bottom left: elevation profile obtained from the DTM relative to the red line transect crossing the RCH in the bottom-right panel

We mapped RCHs by coupling a LiDAR-derived digital terrain model (DTM) and object-based image analysis, comprising image segmentation, feature extraction, and classification (Fig. 2). Airborne LiDAR data were acquired in October 2020 and had an average point density of 15 points m^−2^, resulting in a DTM at 0.5 m spatial resolution. Local slope variability represents critical information for mapping RCHs (Witharana et al. 2018) as they are characterised by a flat platform with a circular or elliptical shape surrounded by upper and lower lips having 1–2 m width and high local slopes. We first derived the slope from the DTM (Fig. 2a, b) and then applied a morphological filter based on the closing procedure, which involves dilation followed by erosion (Soille 2004), using a disc of 7-pixel (3.5 m) in diameter as a structuring element (Fig. 2c). Specifically, we tested structuring elements with a diameter between 5 and 10 pixels and chose 7 pixels as this dimension conformed well with the smallest RCHs. Morphologically closed slope exhibited increased homogeneity within RCHs and greater geometric regularity of their shape compared to unfiltered values, thus, enhancing the automatic delineation of RCHs in the segmentation step (Fig. 2d). While the high spatial resolution of the slope raster derived from the DTM (0.5 m) typically allowed us to recognise RCHs, alterations caused by surface processes like erosion or very dense tree canopy cover posed some challenges to the automatic segmentation of RCHs. We performed image segmentation using the “Seeded Region Growing” algorithm implemented in SAGA (version 7.9.0). We then extracted several features related to topography, shape indices, spectral, textural, and geographic information (Table S1). In particular, for each raster layer, we computed the following set of summary statistics relative to each image object: minimum, maximum, range, sum, mean, variance, standard deviation, Gini coefficient, and percentiles (0.05, 0.25, 0.50, 0.75, and 0.95). We classified image objects through a supervised classification based on the Random Forest (RF) algorithm implemented in the R package “ranger” (Wright and Ziegler 2017). We used 665 objects comprising 212 ground-validated RCHs and 453 visually interpreted non-RCHs as training and validation samples. We mapped each RCH using a sub-metric GNSS device. We computed the user's, producer's, and overall accuracies and the Cohen’s Kappa of the RF classifier by averaging their values obtained during each iteration of a fivefold cross-validation procedure.

### Anthracological analysis

During a field campaign between May 2020 and April 2021, we collected and classified approximately 400 charcoals from 125 previously detected relic charcoal hearths to reconstruct historical forest species composition at Mont Avic. For each RCH, we extracted a variable number of charcoals (2–6 pieces of at least 1 cm^3^) from ~ 1 L of soil obtained from three randomly distributed 10 × 10 × 10 cm excavations. We split charcoal samples to obtain fresh fracture planes oriented along the transverse, longitudinal radial, and longitudinal tangential directions. We used an Olympus BX51 incident light microscope to observe each sample and taxonomically identify the most accurate possible taxon with published references (Schweingruber 1990). We did not perform charcoal radiocarbon dating since the method is not precise for samples more recent than the Industrial Revolution due to the Suess effect (Keeling 1979). For this reason, we assumed the age of the charcoal samples ranging from 220 to 320 years, corresponding to the activity period (1700–1800) of the blast furnaces at Mont Avic (Castello and Paganone 2016).

### Environmental and anthropogenic drivers of forest structure

We sampled the current forest structure and composition by harmonising a previous forest inventory (Cremonese et al. 2007) with a recent (June—October 2020) field campaign. We randomly established and mapped 267 circular plots with a 15-m radius with a GNSS metric device, obtaining an average density of 1 plot per 6 ha of forest (Fig. 1). In each plot, we surveyed trees having a minimum diameter of 7.5 cm and collected the following attributes: tree species, dbh (diameter at the breast height), canopy, and ground cover (bare soil, rocks, litter, forest regeneration, herbs, shrubs). We explored the species diversity of each plot through the Brillouin index based on the basal area per ha of each tree species. The Brillouin index allows describing the variability in woody species abundance measured as the number of individuals (Brillouin 1956; Magurran 2004). We derived several environmental variables from the DTM in a GIS environment, such as terrain roughness (Riley et al. 1999), heat load index (HLI) or the incident radiation of the sun according to the aspect (McCune et al. 2002), topographic wetness index (TWI) as a proxy for moisture accumulation, and topographic position index (TPI) indicating the position of a cell according to the neighbours. We extracted the Euclidean distance of each forest pixel from anthropic features such as RCHs, roads, and buildings as a raster dataset in a GIS environment. In particular, we retrieved georeferenced data for roads and building from the OpenStreetMap database (available at https://www.openstreetmap.org). These data included recent and abandoned buildings, driveways, and hiking trails.

### Data analysis

We derived geometrical attributes such as perimeter, surface area, perimeter/area fraction, shape indices, and density of RCHs in a GIS environment from the object-based classification. We employed descriptive statistics and regression analyses to explore and describe the logic adopted by charcoal burners in the past to locate and build their charcoal kilns in the landscape.

We transformed species abundance measures such as charcoal counts (historical data) and basal area values (current data), obtaining the proportion of each species per survey unit (RCHs and plots). This preprocessing method was necessary to compare historical and current species composition at Mont Avic. Starting from historical and current species proportions, we adopted a simple geostatistical approach to convert our point data to surface data to compare forest composition changes spatially. Our datasets consisted of three coordinates: two geographic (*x*, *y*) and one weight (*z*, species proportion). To obtain raster data from point data, we adopted the Inverse Distance Weighting (IDW), a deterministic interpolation method known to be a conservative estimate of the original data (Bartier and Keller 1996). We obtained the IDW rasters through the Spatial Analyst Tool of ArcGIS v10 software by selecting the following parameters: cell size = 10 m; Power = 2; search radius = variable with 50 points. We assessed the accuracy of each spatial interpolation IDW model by computing common statistical indicators such as mean error (ME), mean absolute error (MAE), root-mean-square error (RMSE), and the regression coefficient (R^2^) of predicted (modelled) against measured (actual) values.

We assessed the variability of current forest structure/composition at the landscape scale (11 variables × 267 plots) in relation to environmental drivers (15 variables × 267 plots) (Table 1) through redundancy analysis. Redundancy analysis is an extension of principal component analysis and was used to investigate the variability explained by the explanatory variables and their correlation with forest structure/composition variation. We performed redundancy analysis using the PC-ORD v7 statistical software. We tested the statistical significance of all ordination analyses by the Monte Carlo permutation method based on 10 000 runs with randomised data.

**Table 1:** List of the 11 forest structure and composition descriptors of 267 plots at Mont Avic, followed by the 15 environmental (anthropogenic, climatic, soil, topographic) independent variables used in RDA

Forest descriptors	Code	Unit	Data source
Density of trees per ha	tph	n ha^−1^	Field surveys
Basal area per ha	ba	m^2^ ha^−1^	Field surveys
Average diameter at the breast height	dbh	cm	Field surveys
Per cent canopy cover	tcd	%	Sentinel-2
Basal area per ha of *P.uncinata*	piun	m^2^ ha^−1^	Field surveys
Basal area per ha of *P.sylvestris*	pisy	m^2^ ha^−1^	Field surveys
Basal area per ha of *L. decidua*	lade	m^2^ ha^−1^	Field surveys
Basal area per ha of *F. sylvatica*	fasy	m^2^ ha^−1^	Field surveys
Basal area per ha of *P.abies*	piab	m^2^ ha^−1^	Field surveys
Basal area per ha of other broadleaves	otbr	m^2^ ha^−1^	Field surveys
Brillouin diversity index	brill	-	Field surveys

Environmental variables	Code	Unit	Data source
Euclidean distance from RCHs	Rch_d	m	LiDAR + GIS
Kernel density of RCHs	Rch_k	–	LiDAR + GIS
Moving coast from buildings	movCo	–	LiDAR + GIS
Euclidean distance from roads	road	m	GIS
Euclidean distance from buildings	build	m	GIS
Elevation	elev	m a.s.l	DEM
Slope	slope	°	DEM
Terrain roughness	roug	–	DEM
Heat load index	hli	–	DEM
Topographic wetness index	Twet	–	DEM
Topographic position index	Tpi	–	DEM
Topographic Roughness Index	Tri	–	DEM
Soil pH	pH	–	VdA soil map
Soil organic carbon	Corg	–	VdA soil map
Soil C-N ratio	C_N	–	VdA soil map

## Results

### Relic charcoal hearths distribution and characteristics

**Fig. 3:**
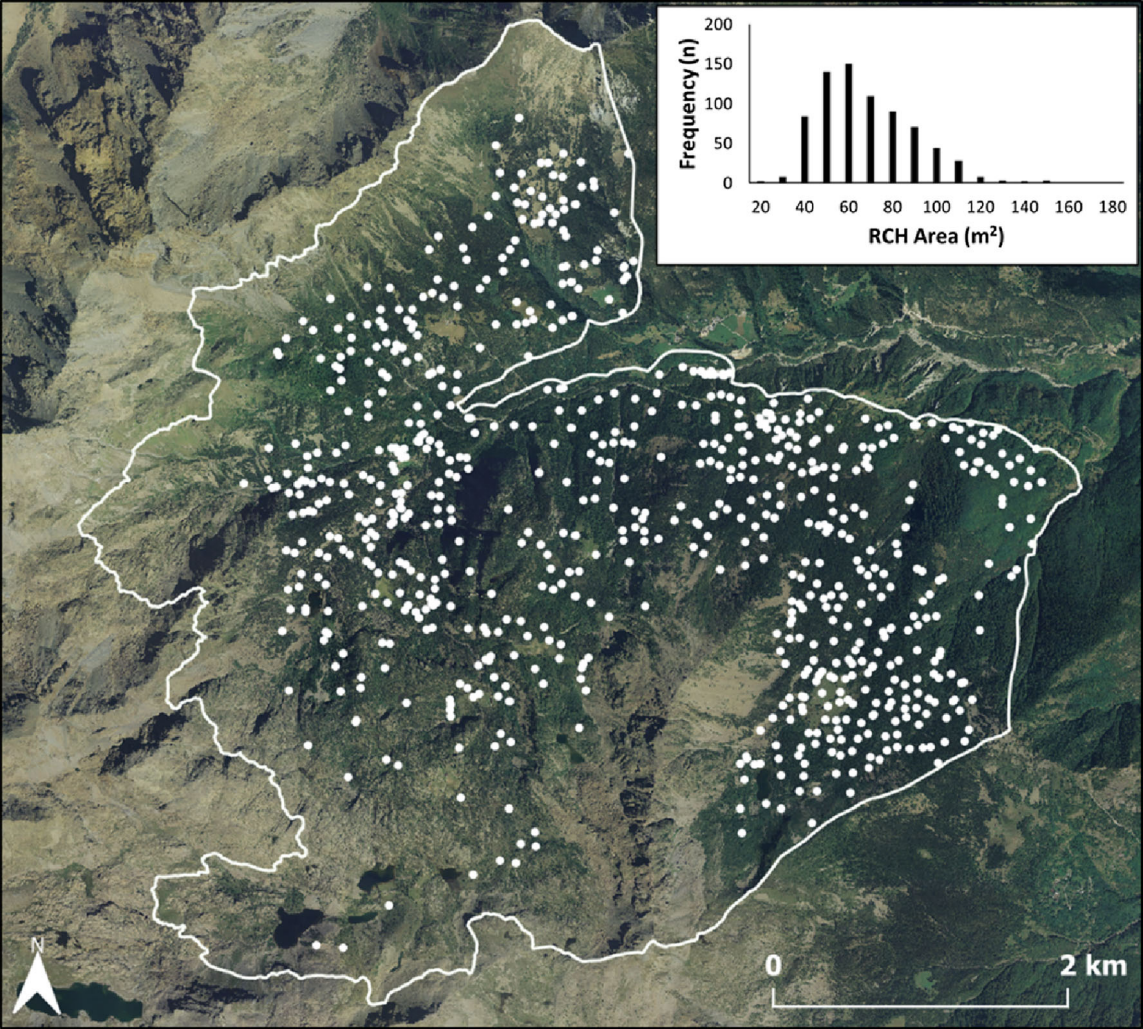
Relic charcoal hearts (RCHs) mapped at Mont Avic, with the frequency distribution of RCHs surface area (upper right inset graph)

We detected 744 RCHs (density = 0.5 ha^−1^; Fig. 3) from the supervised classification based on the RF algorithm, obtaining an overall accuracy of 91.9% and a Cohen’s Kappa statistic of 0.81 (Table S2).

The surface area ranged from 20 to 170 m^2^ with an average value of 63.2 m^2^ and a positively asymmetric distribution (Fig. 3, inset). RCHs showed an average simple elliptical shape expressed by a low perimeter/area ratio (0.64), similar to a circle of the same surface area (0.45). RCHs were located almost everywhere, with elevations ranging from 1088 to 2287 m a.s.l., closer to roads (172.4 m) than buildings (451.9 m). A weak and positive relationship (Pearson’s *r* = 0.35) exists between RCHs area and elevation and a negative one (Pearson’s *r* = − 0.36) with slope.

### Forest composition change and human legacy

**Fig. 4:**
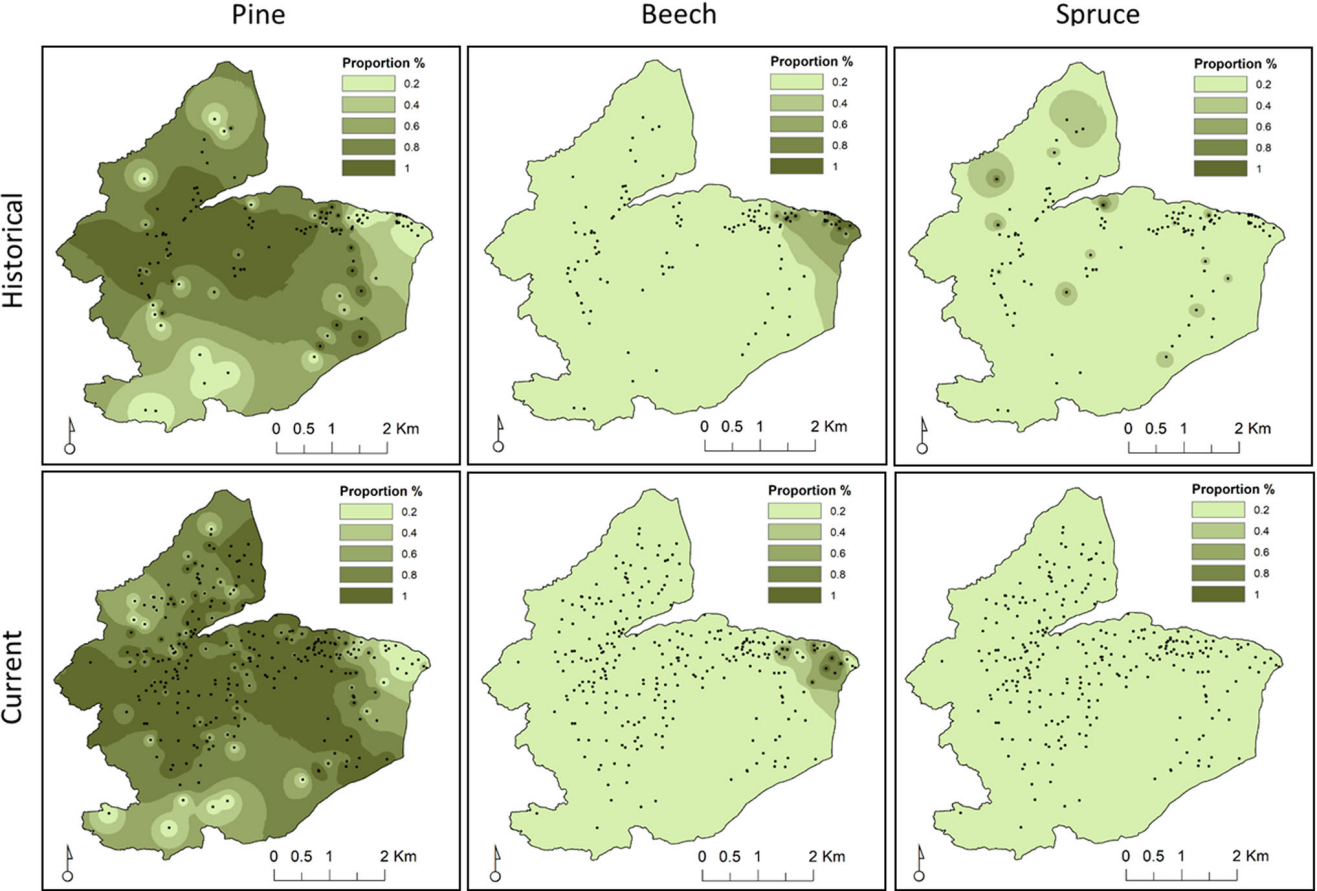
Historical (pedoanthracological data *n* = 125) and current (field surveys *n* = 267) forest composition expressed as spatially interpolated (IDW) surface data

From the classification of approximately 400 charcoals, we obtained a picture of the historical forest species composition where *Pinus* dominated (62%), followed by *Fagus* (15%), *Picea* (8%), and a group of indistinguishable *Larix/Picea* samples (13%). Regarding the distinction between Picea and Larix, specimens were attributed to the former only in the presence of an evident gradual transition between earlywood and latewood. From a comparison between the current and historical overall species composition, we observed an increase of *Pinus* (75%) and *Larix* (18%) to the detriment of *Fagus* (5%) and *Picea* (0.003%). By interpolating our point dataset, we obtained a spatially explicit comparison between the current and historical species composition of the three main groups. The more evident change is related to the distribution of the *Pinus* group that invaded a larger surface area at the expense of spruce and beech groups (Fig. 4). The accuracy of the current IDW maps was consistently higher (RMSE < 1.8%) than the historical ones (RMSE < 4.8%), and *Abies* performed better than *Pinus* and *Fagus* (Table S3).

### Drivers of current forest structure

**Fig. 5:**
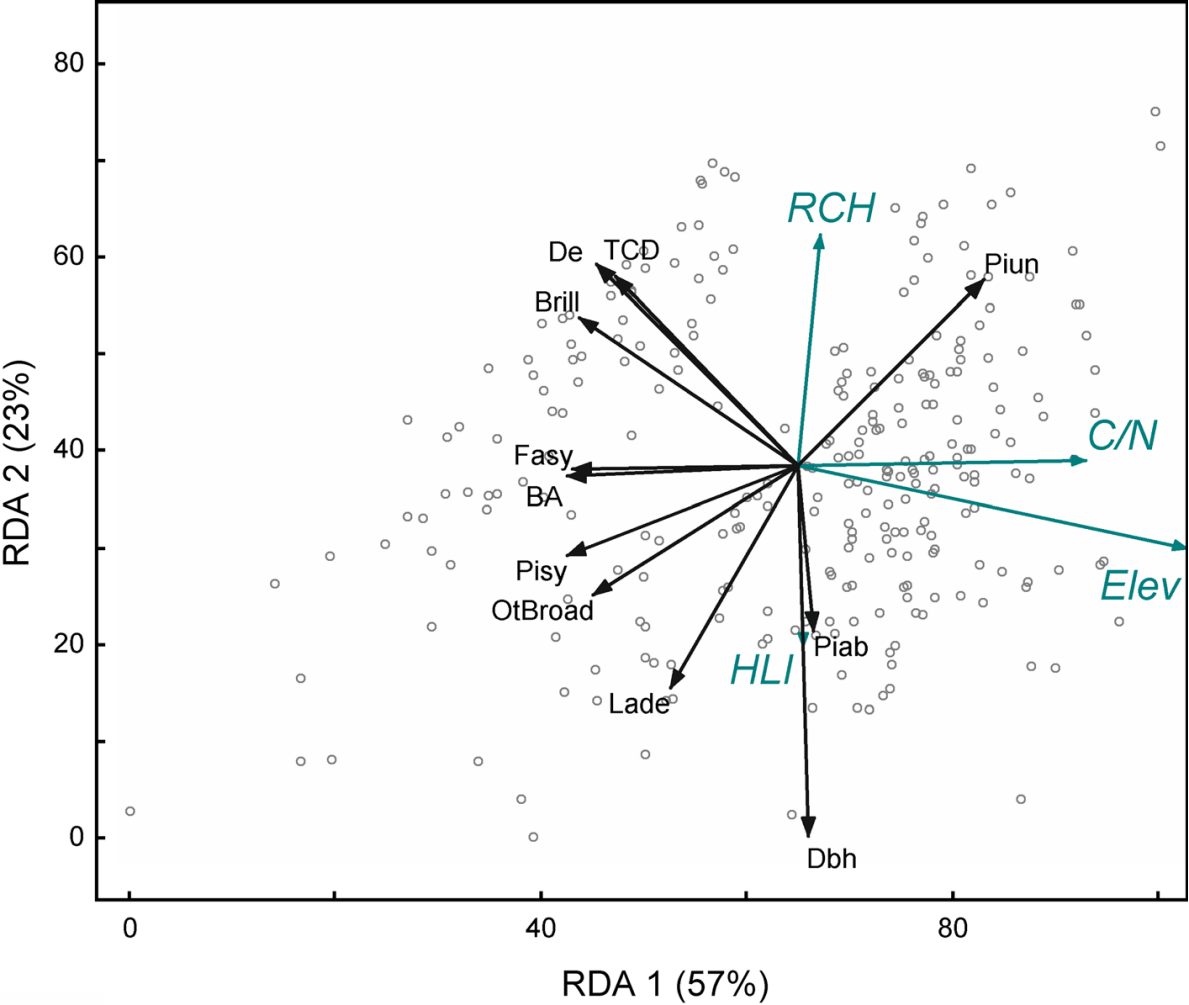
Redundancy analysis (RDA of 267 plots) of forest structure in relation to environmental characteristics at landscape scale. Black arrows are the forest structure variables (De: trees per ha; BA: basal area per ha; Dbh: average diameter at the breast height; TCD: tree cover density or canopy cover; Brill: Brillouin diversity index; Fasy: basal area of *Fagus sylvatica*; Pisy: basal area of *Pinus sylvestris*; Piun: basal area of *Pinus mugo* subsp. *uncinata*; Lade: basal area of *Larix decidua*; Piab: basal area of *Picea abies*; OtBroad: basal area of other broadleaved trees). Cyan arrows represent the “biplot scores of environmental variables” (Elev: elevation; HLI: heat load index; C/N: Carbon-to-Nitrogen ratio in soil; RCH: density of relic charcoal hearths)

We analysed the influence of environmental and anthropogenic factors on the structure of Mont Avic forests through direct gradient analysis. The first and second axes accounted for 57.3 and 22.6% of the total variation, respectively (Fig. 5; Table S4). Dense stands dominated by mountain pine were positively associated with the density of RCHs. In the opposite gradient, on warmer sites, we found a significant presence of European larch in open stands with bigger trees. Conversely, productive and more diverse stands with European beech and Scots pine were located at lower elevations and negatively associated with C/N soil content.

## Discussion

The history of charcoal kilns and their relationships with forest ecosystem structures and functions is a highly multidisciplinary topic. Still, the integration of tools and heterogeneous data sources is rarely found in the literature. In this sense, our research at Mont Avic proposes a novel approach that integrates different datasets and disciplines (field forest structure, anthracological, LiDAR remote sensing, and GIS) based on their spatial attributes.

### Relic charcoal hearths distribution and characteristics

The object-based automatic classification of LiDAR-derived DTM allowed us to detect an average density of 0.5 ha^−1^ charcoal platforms, lower than that reported for other European forest landscapes. For example, 1.2 ha^−1^ RCHs in central Tuscany, Italy (Biondi et al. 2021), 1.5 ha^−1^ in the Black Forest, Germany (Ludemann 2010), 1–3 ha^−1^ in Wallonia, Belgium (Hardy et al. 2016). In North America, the density of RCHs was similar (0.1–0.3 ha^−1^) in NW Connecticut (Johnson and Ouimet 2021) but higher (1 ha^−1^) in New England (Witharana et al. 2018). The lower density of RCHs found at Mont Avic is probably related to the limited biomass productivity of conifer forests growing on mafic and ultramafic parent materials (D’amico et al. 2008; Kim and Shim 2008).

The shape of RCHs found at the Mont Avic study site was elliptical/circular, as observed in several other charcoal production sites (Johnson et al. 2015; Raab et al. 2017; Witharana et al. 2018), but the average size (63 m^2^) was bigger than other platforms (22–55 m^2^) detected in broadleaved forests of the Italian Apennines (Carrari et al. 2016). We found RCHs close to trails, roads, and water sources, with a higher abundance in moderate slope sites. This was probably because trails connected each charcoal kiln, and the biomass productivity of flat areas was high. The contemporary presence of canopy cover and the proximity to ancient roads are also common in other sites.

### Human legacies on forest composition

As a result of the intense historical charcoal production at Mont Avic, conifer light-demanding species including mountain pine, Scots pine, and European larch expanded to the detriment of late seral species such as Norway spruce and European beech. This trend is evident even considering that it is not always possible to anatomically distinguish between spruce and larch wood charcoals (Schweingruber et al. 1978; Wright, 2017). Current forest composition results from a relatively short-rotation (30–40 years) clearcut system that periodically increased the availability of light to the understory, favouring pioneer species and the homogenisation of forest structure. Moreover, the uniform spatial distribution of RCHs within our study area reveals intense exploitation of the entire watershed for charcoal production. Our spatially explicit analyses demonstrated that the highest density of relic charcoal hearths is currently associated with pure mountain pine stands. An expansion of early seral species in forests heavily exploited for wood charcoal production has been observed in many areas. Some examples can be found in Germany, where birch dominated the secondary succession (Nelle 2003), in the Slovak Karst National Park, where oak trees partially replaced European beech due to intense coppicing (Máliš et al. 2021), and in Axial Pyrenees, where mountain pine and Scots pine were also favoured (Pèlachs et al. 2009).

Archaeological evidence of charcoal production from conifer forests in the Italian Alps is limited to the central (Castelletti et al. 2012) and eastern Alps (Backmeroff 2001). However, the use of conifer species for charcoal production has been documented in many European countries (e.g. Rutkiewicz et al. 2017). A reduced species diversity due to the historical ‘charcoal silviculture’ is an enduring legacy that will likely drive the future dynamics of forest ecosystems in human-dominated landscapes (Garbarino and Weisberg 2020). Many ecological niches hosting a wide variety of plants and animals are usually found in forest ecosystems expressing a rich species composition (e.g. Lefsky et al. 2002). Forest species diversity also guarantees a reduced risk of biotic disturbances (Haas et al. 2011) and enhances the post-disturbance resilience of forest ecosystems (e.g. Cadotte et al. 2011).

### Drivers of current forest structure

Big larches with a sporadic presence of Norway spruce dominate warmer sites historically occupied by pastures and larch wood pastures (Anselmetto et al. 2021). Conversely, a high RCH concentration was positively associated with dense stands of small-diameter mountain pine trees on cooler sites. The latter is likely the result of extensive and frequent clearcut harvesting that favoured a homogeneous cohort of young pine stands to the detriment of forest diversity.

Several authors documented a simplification of forest structure due to historical charcoal production, and nowadays, it represents a particular issue in some regions of the world, such as Africa (e.g. Gardner et al. 2016; Kiruki et al. 2017). Marginal and remote sites, generally characterised by steep slopes on the north-facing aspect, are currently dominated by conifer forests exhibiting a relatively high density of young trees. These forest landscapes, saved from logging during the mediaeval times, exhibit a strongly simplified structure due to short-rotation clearcut to produce wood charcoal during the 1700–1800 (Giordano 1864).

Other authors highlighted that multitaxon species richness is strongly affected by the clearcut system with a substantial decline in biodiversity (Paillet et al. 2010). Therefore, a rewilding process of marginal protected forest landscapes should be favoured to increase species diversity and structural complexity required by forest specialist vertebrates that are still rare in these environments (e.g. *Glaucidium passerinum* and *Picoides tridactylus*; Baroni et al. 2020; Keller et al. 2020).

## Implications for historical landscape ecology and management

Historical ecology provides essential information to evaluate the resilience of an ecosystem to past human disturbances, acting as conservation, restoration, and management tool (Beller et al. 2020). Shedding some light on local forest history aids in revealing the reference ecosystem for future restoration activities (Whitlock et al. 2018). The multidisciplinary approach presented here can be replicated in many other forest landscapes to detect and describe the main land-use legacies. Moreover, stressing the spatial component of data allows a broader understanding of the human-nature relationship in forest ecosystems.

Marginal lands, such as the European Alps, host great biodiversity and an abundance of rare and sensitive species and are frequently associated with protected areas (Hazen and Hatamatten, 2004). Given the historical land-use, gentler and warmer slopes were usually dominated by pastures and crops (Kulakowski et al. 2011; Garbarino et al. 2013), while steeper and cooler ones were devoted to wood and charcoal production (Giordano 1864). Therefore, quantitative knowledge of complex social-ecological ecosystems with an intense land-use history is fundamental to manage protected marginal areas by planning specific nature-based solutions.

## Supplementary Information

Below is the link to the electronic supplementary material.

Supplementary file1 (PDF 571 kb)
