# Supplementary material for: Legacy of wood charcoal production on subalpine forest structure and species composition
Source: Ambio. 2022 Jun 9;51(12):2496–507. doi: 10.1007/s13280-022-01750-y (PMC9584004; doi:10.1007/s13280-022-01750-y)
Supplement: Supplementary file 1 — Supplementary file1 (PDF 571 kb) [file 13280_2022_1750_MOESM1_ESM.pdf]

Ambio

Supplementary Information

This supplementary information has not been peer reviewed.

Title: **Legacy of wood charcoal production on subalpine forest structure and species composition**

Table S1. List of features extracted for each object related to topography, shape, spectral and textural characteristics.

Type	Name	Description	Source
Topography	DTM	LiDAR-derived digital terrain model at 0.5 m resolution	Airborne LiDAR
	Slope	DTM-derived at 0.5 m resolution	SAGA “Tool Slope, Aspect, Curvature”
	TPI	Topographic Position Index	SAGA “Module Topographic Position Index (TPI)”
	TRI	Topographic Ruggedness Index	SAGA “Module Terrain Ruggedness Index (TRI)”
Shape indices	A	Area	SAGA “Tool Polygon Shape Indices”
	P	Perimeter	
	P/A	Interior edge ratio	
	P/sqrt(A)	Interior edge ratio 2	
	Deqpc	Equivalent projected circle diameter (=2*sqrt(A/pi))	
	Sphericity	Ratio of the perimeter of the equivalent circle to the real perimeter (=P/(2*sqrt(A*pi)))	
	Shape Index	Inverse of the spericity	
	Dmax	Maximum diameter calculated as maximum distance between two polygon part's vertices	
	DmaxDir	Direction of maximum diameter	
	Dmax/A	Max diameter/Area	

	Dmax/sqrt(A)	Max diameter/Area 2	
	Dgyros	Diameter of gyration, calculated as twice the maximum vertex distance to its polygon part's centroid	
	Fmax	Maximum Feret diameter	
	FmaxDir	Direction of the maximum Feret diameter	
	Fmin	Minimum Feret diameter	
	FminDir	Direction of the minimum Feret diameter	
	Fmean	Mean Feret diameter	
	Fmax90	Feret diameter measured at an angle of 90 degrees to that of the Fmax direction	
	Fmin90	Feret diameter measured at an angle of 90 degrees to that of the Fmin direction	
	Fvol	Diameter of a sphere having the same volume as the cylinder constructed by Fmin as the cylinder diameter and Fmax as its length	
Spectral information	R, G, B bands	Very-high resolution (0.5 m) satellite imagery	Google Earth
Textural information	Closing; morphological gradient	Mathematical morphology operations performed using an elliptical structuring element with a diameter of 7 and 9 pixels	SAGA “Morphological Filter (Open CV)”

	Contrast; energy; entropy; variance	Local statistical measures computed using a circular kernel with diameter of 11, 21 and 41 pixels	SAGA "Local Statistical Measures"
	Angular Second Moment; contrast; correlation; variance; inverse difference moment; sum average; sum entropy; entropy; difference variance; difference entropy; measure of correlation-1; measure of correlation-2	Haralick textures computed with a kernel having a diameter of 11 and 21 pixels and in all directions.	SAGA "Textural Features"
Geographic information	X and Y coordinates	Coordinates relative to the centroids of each image object	Image objects

Table S2. Confusion matrix relative to the classification of RCHs obtained from a 5-fold cross validation procedure. Rows contain true classes, columns predicted classes. Producer's (PA), user's (UA) and overall accuracies relative to the classification of image objects obtained using a 5-fold cross-validation procedure are indicated in the last two columns.

**Class**	**RCH**	**Non-RCH**	**Total**	**PA**	**UA**
**RCH**	173	39	212	92.0	81.6
**Non-RCH**	15	438	453	91.8	96.7
**Total/Overall**	188	477	665	91.9	

Table S3. Statistical indicators (RMSE = Root Mean Square Error; ME = Mean Error; MAE = Mean Absolute Error; R^2 = regression coefficient) to assess IDW interpolating model performances.

IDW Model	RMSE	ME	MAE	R^2
Pinus Hist	0.0373	0.0024	0.0127	0.9928
Pinus Curr	0.0177	0.0013	0.0037	0.9969
Fagus Hist	0.0478	-0.0057	0.0113	0.9740
Fagus Curr	0.0015	0.0000	0.0004	0.9999
Abies_Hist	0.0174	0.0015	0.0049	0.9927
Abies_Curr	0.0007	0.0000	0.0001	0.9995

Table S4. Statistical overall descriptors of the redundancy analysis (RDA) performed on 267 plots at MA study area (first 5 rows) and inter-set correlations between RDA axis and five predictors (last 5 rows).

	RDA-1	RDA-2	RDA-3
Eigenvalue	6.307	2.481	1.537
% of variance explained	57.30	22.60	14.00
Cumulative % explained	57.30	79.90	93.90
Pearson Correlation	0.539	0.350	0.187
Kendall Correlation	0.400	0.256	0.144
RCH	-0.015	0.334	-0.060
Slope	-0.216	-0.177	0.021
Elevation	0.465	-0.150	-0.002
HLI	0.004	-0.166	-0.156
C/N	0.346	0.001	-0.058
